# Plasma-Mediated Inactivation of *Pseudomonas aeruginosa* Biofilms Grown on Borosilicate Surfaces under Continuous Culture System

**DOI:** 10.1371/journal.pone.0108512

**Published:** 2014-10-10

**Authors:** Kurt G. Vandervoort, Graciela Brelles-Mariño

**Affiliations:** 1 Physics and Astronomy Department, California State Polytechnic University, Pomona, California, United States of America; 2 Biological Sciences Department, California State Polytechnic University, Pomona, California, United States of America; University of Technology Sydney, Australia

## Abstract

Biofilms are microbial communities attached to a surface and embedded in a matrix composed of exopolysaccharides and excreted nucleic acids. Bacterial biofilms are responsible for undesirable effects such as disease, prostheses colonization, biofouling, equipment damage, and pipe plugging. Biofilms are also more resilient than free-living cells to regular sterilization methods and therefore it is indispensable to develop better ways to control and remove them. The use of gas discharge plasmas is a good alternative since plasmas contain a mixture of reactive agents well-known for their decontamination potential against free microorganisms. We have previously reported that *Pseudomonas aeruginosa* biofilms were inactivated after a 1-min plasma exposure. We determined that the adhesiveness and the thickness of *Pseudomonas* biofilms grown on borosilicate were reduced. We also reported sequential morphological changes and loss of viability upon plasma treatment. However, the studies were carried out in batch cultures. The use of a continuous culture results in a more homogenous environment ensuring reproducible biofilm growth. The aim of this work was to study plasma-mediated inactivation of *P. aeruginosa* biofilms grown on borosilicate in a continuous culture system. In this paper we show that biofilms grown on glass under continuous culture can be inactivated by using gas discharge plasma. Both biofilm architecture and cell culturabilty are impacted by the plasma treatment. The inactivation kinetics is similar to previously described ones and cells go through sequential changes ranging from minimal modification without loss of viability at short plasma exposure times, to major structure and viability loss at longer exposure times. We report that changes in biofilm structure leading to the loss of culturability and viability are related to a decrease of the biofilm matrix adhesiveness. To our knowledge, there has been no attempt to evaluate the inactivation/sterilization of biofilms grown in a continuous system.

## Introduction

In the past, the microbial world was thought as composed of isolated microorganisms growing apart. However, this model is more the exception than the rule. Most microbes are “social” and prefer to live and thrive as part of communities where interactions take place [1]. A biofilm is an example of this type of community where cooperative effects become important. Biofilms are microbial communities that grow attached to a surface and embedded in a viscous matrix composed of exopolysaccharides together with proteins and excreted nucleic acids. Biofilms are present almost everywhere and impact all aspects of our life. Not all bacterial biofilms are detrimental but in many cases their presence is responsible for expensive and undesirable effects such as disease, prostheses colonization, product contamination, biofouling and equipment damage, pipe plugging, tooth decay, and dental plaque. About 90 percent of infections in humans and 65 percent of nosocomial infections are due to biofilms according to the National Institutes of Health (NIH) and The Center for Disease Control (CDC) respectively. Biofilms also contaminate water sources and cause pipe plugging. *Helicobacter pylori*, a microorganism responsible for gastric ulcers, have been found in pipes in drinking water systems. Therefore not only is the industrial contamination a concern but also the possibility of spreading disease by contaminated water [2].

Studies on microbial growth and its control using free-living, planktonic microorganisms have provided a good deal of information. However, these results cannot always be easily extrapolated to microbial communities such as biofilms, more resilient to standard killing methods. The use of moist heat in the autoclave is still an inexpensive method for many applications but it cannot be applied to all situations, such as prosthetic devices or thermosensitive materials. Low-temperature disinfection can be achieved using chemicals; among these, ethylene oxide is both mutagenic and carcinogenic and chlorine pose an environmental hazard and risks to human health. Radiation can be used in some but not all the cases. Therefore, it is indispensable to develop better ways to control and remove biofilms. The use of gas discharge plasmas is a good alternative since plasmas contain a mixture of reactive species, free radicals, and UV photons well-known for their decontamination potential against free microorganisms [3]. Investigations aimed at elucidating the effects of plasmas on bacterial cells were initiated, mostly in the United States since the mid nineties [4]. Most of those studies were carried out with microorganisms in the free-living state or with spores. By the middle of the last decade, our group and a few others reported the use of plasma for biofilm disinfection or inactivation [5]–[9].

More recently we reported the use of plasma to inactivate *Pseudomonas aeruginosa* biofilms [10]. *P. aeruginosa* is a Gram-negative opportunistic pathogen that preys on victims with compromised immune systems, patients on respirators, and causes infections of burned tissue and colonization of catheters and medical devices. It also co-colonizes, together with *Burkholderia cenocepacia*, lung tissue and is the main cause of mortality in cystic fibrosis patients [11]. *Pseudomonas* biofilm inactivation/sterilization have been intensively studied by different approaches such as the use of biocides, antibiotics or a combination of both [12], [13]; the use of chelators [14]; and compounds such as furanone and *N*-acyl homoserine lactones [15], [16]; and the modification of surfaces [17], [18], among others.

In our previous contributions, *P. aeruginosa* biofilms were grown on borosilicate, polycarbonate, and staineless-steel surfaces in batch culture. We showed that almost 100% of the cells were inactivated after a 5-min plasma exposure. Through atomic-force-microscopy (AFM) we determined that the adhesiveness to borosilicate and the thickness of the *Pseudomonas* biofilms grown on borosilicate were reduced and we reported sequential morphological changes and loss of viability upon plasma treatment [10], [19]. However, all the above studies were carried out with biofilms grown in a batch culture system. The way biofilms grow in nature differs from the way they are grown in batch in the laboratory. A batch culture is a closed system starting with an inocculum that grows until nutrients are depleted or toxic products accumulate in the reactor. Therefore, bacterial concentration varies with time. A continuous culture is a way of growing microorganisms in which there is a continuous flow of nutrients in the environment and growth does not depend on time as a variable. In nature, biofilms are surrounded by an aqueous environment in an open system and therefore the continuous culture better mimicks biofilm growth in the real world. The use of the continuous culture results in a more homogenous environment ensuring reproducible biofilm growth.

In this paper we present data on plasma-mediated inactivation of *P. aeruginosa* PAO1 biofilms grown on borosilicate in continuous culture. We show that the inactivation kinetics is similar to previously described ones [10], [19] and that cells go through sequential changes ranging from minimal modification without loss of viability at short plasma exposure times, to major structure and viability loss at longer exposure times. We report that changes in biofilm structure leading to the loss of culturability and viability are related to a decrease of the biofilm matrix adhesiveness. To our knowledge, there has been no attempt to evaluate the inactivation/sterilization of biofilms grown in a continuous system.

## Materials and Methods

### A. Biofilm growth

*Pseudomonas aeruginosa* strain PAO1 biofilms were produced in continuous culture using the CDC biofilm reactor (BioSurface Tech., MT) until constant optical density for 24 hours. The biofilms were grown on borosilicate (glass) coupons in TSB (Tryptic Soy Broth) at 37°C with agitation. After the selected growth time, the coupons were aseptically removed from the reactor and unbound bacteria were removed by rinsing the coupons twice with sterile saline. Coupons were air-dried prior to being subjected to gas discharge plasma for various exposure times (1, 2, 3, 5, 15, and 30 minutes) under sterile conditions. A control without plasma treatment (0-min exposure time) was included. Coupons were placed in a wet chamber after treatment and incubated with 50 *µ*L of sterile saline for 10 minutes. Biofilms were then scraped off and suspended in 1 mL of sterile saline, serially diluted, and suspensions were plated in duplicate on TSA (Trypctic Soy Agar) medium. Plates were incubated at 37°C and evaluated for colony-forming-units (CFU) formation by counting the colonies.

### B. Plasma Generation and Conditions

Atmospheric-pressure gas discharge plasma was produced using a commercially available inductively-coupled Atomflo 300 reactor (Surfx Technologies, CA) that delivers a plasma jet [20]. The reactor consists of two perforated rectangular plates separated by a gap 1.6-mm across. The upper aluminum electrode is connected to a 100-W RF power supply (13.56 MHz), and the lower electrode is grounded. The size of the plasma showerhead is 0.63-cm wide by 2.54-cm across. For the experiments, an atmospheric-pressure plasma jet was generated using a He flow of 20.4 L/min, a secondary gas flow (N_2_) of 0.135 L/min, and an input power of 35 W. Both gases were industrial grade. The plasma applicator was mounted such that the showerhead was 4 mm away from the biofilm.

### C. Temperature determination

Glass coupons were exposed to plasma to test the temperature reaching the coupon during plasma treatment. A thermometer was placed directly on the coupon surface and the temperature was monitored and recorded once a minute for 10 minutes.

### D. Atomic Force Microscopy (AFM)

*P. aeruginosa* biofilms were grown on glass coupons, treated with plasma for 0, 1, and 30 minutes, and processed as indicated above. The coupons were rinsed twice, air-dried, and AFM images were obtained in air in contact mode using a Quesant Instruments Universal Scanning Probe Microscope. Commercial silicon cantilevers from MikroMasch were employed with spring constants from 0.1 to 0.5 N/m. For each coupon, at least four widely separated regions were imaged to obtain a representative sample and ensure reproducibility. Images consisted of 500 lines of 500 points per line for a total of 250,000 pixels of data.

To assess micromechanical properties of the biofilm, force-displacement curves were obtained at widely separated locations on the coupon, to achieve representative results. The procedure consisted of bringing the AFM tip in contact with the sample and then moving the sample upward a set distance while monitoring the deflection of the cantilever. At each sampling location where force-displacement curves were obtained, the tip was brought in and out of contact at a rate of 0.5 Hz to the maximum set sample deflection (0.6 µm) and the displacement curve was recorded upon the fifth trial. This technique helped to reduce hysteresis that was often observed in the first few trials. The process was then repeated so that at least five force-displacement curves were recorded at each sampling location. Similar techniques for measuring the micromechanical properties of bacteria and bacterial biofilms have been employed successfully in previous studies by us [10], [19] and by others [21], [22]. For comparing samples with different plasma treatments, all of the force-displacement data were recorded on the same day using the same cantilever. This method ensured control for humidity and cantilever dependent factors (such as spring constant) that can influence the shape of these curves [23].

### E. Virulence Tests

A plant assay was developed to test the virulence of biofilm-derived bacteria treated with plasma for various exposure times [19]. The mid-vein of surface-sterilized leaves of Romaine lettuce was inoculated with a 1 mL-suspension of *P. aeruginosa* cells obtained after treating biofilms with plasma as indicated above and diluted with sterile saline to OD = 1. Unexposed *P. aeruginosa* was used as a positive control. A leaf injected with saline as well as a leaf that had not undergone any injections were also included as controls. Leaves were then aseptically placed into UV sterilized- plastic bags. The bags were sealed, laid flat at 37°C and incubated for three additional days. Tissue damage was assessed and photos of leaves were taken on the fourth day.

## Results and Discussion

### Survival Curves and Inactivation Kinetics

**Figure 1:**
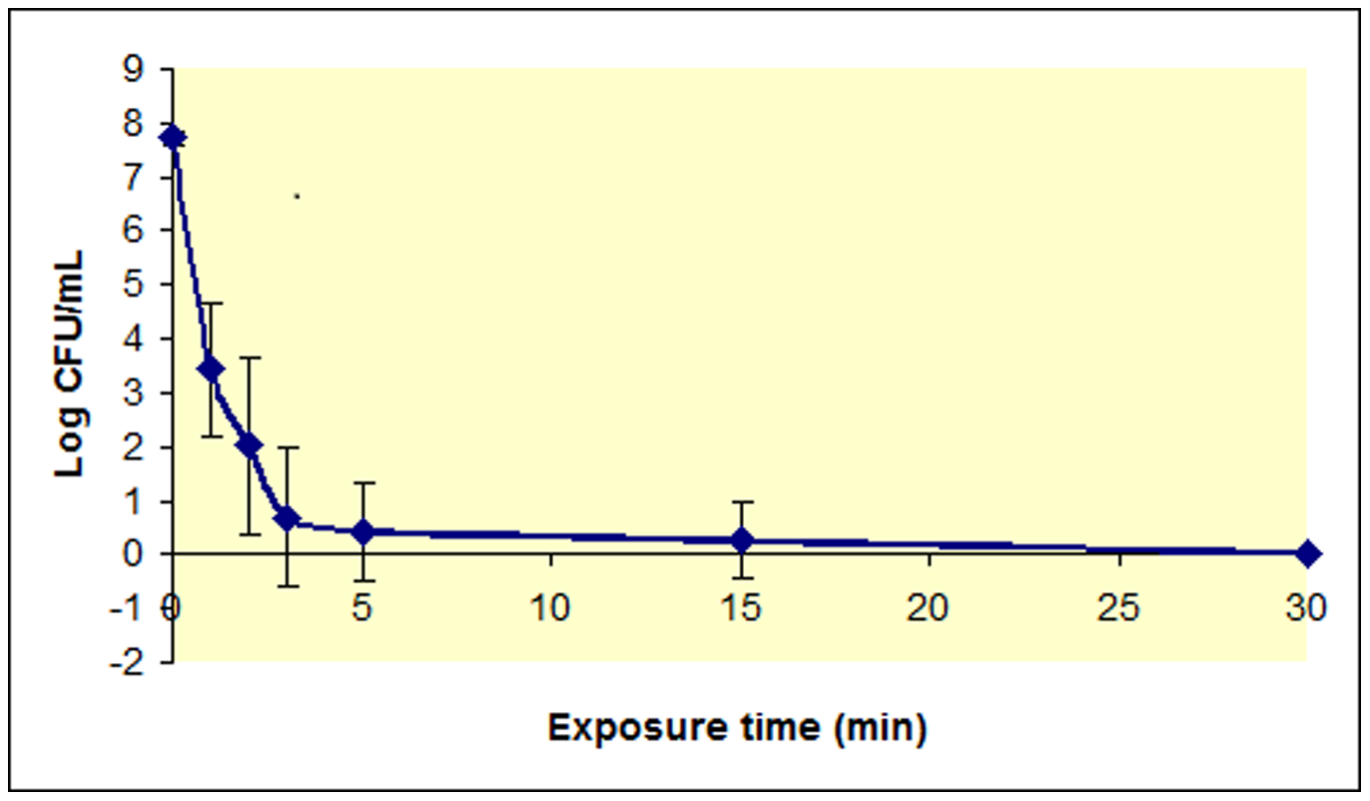
Survivor curve for *P. aeruginosa* plasma-treated biofilms. Log of the number of *P. aeruginosa* CFU/mL vs plasma exposure time (0 to 30 minutes). Results are the average of five independent experiments. Each experiment was performed in duplicate. Bars represent the standard error of the mean.

The concentration of remaining culturable cells versus plasma exposure time for borosilicate-grown *P. aeruginosa* biofilms is shown in Figure 1. At time 0 the concentration of culturable cells corresponds to the control without plasma treatment. The graph shows that *P. aeurginosa* biofilms grown in continuous culture were almost completely removed after a one-minute treatment with plasma. A good measure of the removal efficiency is the determination of the decimal reduction time (D value) [24], [25], the time required to reduce an original concentration of microorganisms by 90%. This parameter has been originally defined for thermal killing of microorganisms by autoclaving. The results show a rapid decline in CFU/mL (first phase) followed by a much slower subsequent decline. As the kinetics of biofilm inactivation shows a double-slope curve, two D values were determined: one for short exposure times (D_1_) and another one for the portion of the curve showing a slower decline (D_2_). D_1_ is 27 sec and D_2_ is 6.13 min. We previously reported double-slope kinetics and D-values of 2.3 min (D_1_) and 37.4 min (D_2_) for *Chromobacterium violaceum* biofilms grown on polyesterene microtiter plates [5]. We also reported double-slope kinetics for *P. aeurginosa* biofilm grown in batch cultures although D values were not calculated [10]. In that previous work, we showed that regardless of the biofilm age, there was a clear decrease in the percentage of surviving cells versus time. Similar results were reported for *C. violaceum* biofilms grown for 4 or 7 days on polystyrene microtiter plates [5], [26]. In the case of *P. aeruginosa* biofilms, the decrease in the percentage of viable cells was more dramatic since there are almost no culturable cells after a 5-minute treatment with plasma. As previously reported for *P. aeruginosa* biofilm of different maturity grown on borosilicate coupons, most of the inactivation occurs before a biofilm exposure to plasma of less than one minute.

To rule out the effect of temperature on the inactivation of the biofilm we measured the temperature of the gas reaching the coupon surface. Equilibrium temperatures of 35 °C were reached within a few minutes and remained constant over time confirming that temperature is not responsible for biofilm inactivation since *P. aeruginosa* is a human pathogen that prefers living a t 37°C.

To rule out effects of gas flow on biofilms, plasma-treated biofilms were compared to biofilms exposed to a flow of gas in the absence of plasma (plasma source turned off) as described elsewhere [26]. Although the flow of gas dried out cells and caused a decrease in the number of CFUs of about 5 to 10%, this decrease is not significant compared to the decrease produced by plasma. Therefore, cell inactivation is due to plasma treatment and not due to excessive cell drying from the gas flow.

Other authors have reported bi and triphasic behaviors for plasma-assisted killing of free-living microorganisms and spores. Two-slope behavior with a smaller D value for shorter exposure times and a higher D value for longer exposure times, were reported by Kelly-Winterberg et al. for *Staphylococcus aureus and Escherichia coli* on polypropylene samples, and by Laroussi *et al*. for *P. aeruginosa* in liquid suspension [27], [28]. The biphasic curves were explained by the damaging of the cell membrane due to plasma reactive species in the first phase. Once the membrane is compromised, the reactive species can easily penetrate causing rapid cell death in the second phase. We first hypothesized that the initial rapid decline of CFU/mL might be due to the killing of the upper layers of microorganisms in the biofilm, that were more exposed to plasma. After this initial killing, plasma has to penetrate layers of cell debris and dead cells before reaching the inner portion of the biofilm [5]. However, no experimental evidence was obtained in our laboratories to support the hypothesis. Further results from our laboratory showed that plasma-mediated biofilm inactivation proceeds through a first step in which bacterial cells are not culturable but still alive, followed by a second step, characterized by a higher D-value, in which cells are killed. Results are consistent with a first step in which bacteria enter a viable-but-non-culturable (VBNC) state and/or they result in spheroplasts that are smaller in size due to damage and further removal of cell walls after plasma treatment. These spheroplasts are non-culturable but still alive since they retain an intact cell membrane [26]. The VBNC state is a dormant state that represents a survival mechanism of bacteria facing one or more environmental stresses which might otherwise be ultimately lethal to the cell and that has been reported for many gram-negative organisms [29]. Regardless of the mechanism, it was clear from our results that treating biofilm cells with plasma for short exposure times resulted in cells that were not culturable but still alive. These findings paved the way to a change in the paradigm that implied that cells were killed based only on their lack of culturability. Since then, we always carried out viability experiments before concluding that bacteria are killed just because we cannot count colonies [26], [10], [19].

### AFM images and force-displacement curves

**Figure 2:**
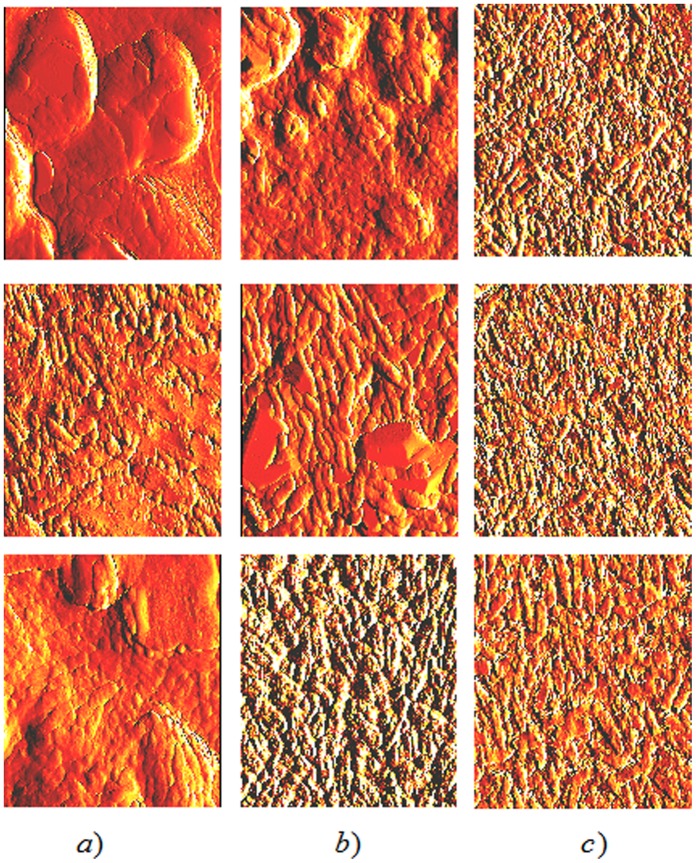
Atomic force microscope images of *P. aeruginosa* biofilms treated with plasma for 0 minute (column a), 1 minute (column b) and 30 minutes (column c). Data for samples 1, 2, and 3 are displayed in the top, middle, and bottom rows, respectively. Image areas are 10×10 µm^2^.

Figure 2 displays a series of AFM images of the biofilm, before and following plasma treatment. The control (0 minute-treatment) images in the left column of Figure 2 show the typical tridimensional structure of a *P. aeruginosa* biofilm. The 1 minute-treated samples in the middle column of Figure 2 depict some morphological changes in the biofilm but still show distinctly discernable bacteria. In the right column of Figure 2, the 30 minute-treated samples display a flatter, more disrupted structure that less convincingly resembles a tridimensional biofilm although there are still discernable bacteria. We have previously reported sequential structural changes of the biofilm after plasma treatment. Those changes ranged from minimal ones at short exposure times to very noticeable modifications at longer exposure times for both *C. violaceum* and *P. aeruginosa* biofilms [10], [19], [26], [30]. We also reported a decrease in biofilm thickness after plasma treatment for *P. aeruginosa* biofilms grown on glass coupons in batch culture [10]. In the case of *Chromobacterium* biofilms, no recognizably intact cells but only debris or cell remnants were obtained after a 60-minute plasma treatment [26], [30]. However, in those experiments cells were dettached from the biofilm by sonication before processing and also, exposure times were longer than the ones used for the experiments reported here. If bacterial envelopes were damaged by plasma treatment, cells might have become weaker and more prone to become disrupted/broken by the sonication treatment. In our present case, cells remain within the biofilm structure and attached to the surface. Also, although both *Chromobacterium* and *Pseudomonas* are gram-negative bacteria, they might exhibit slightly different responses to plasma treatment. Results from this work confirm our previous observations with other types of biofilms and demonstrate that the effect of plasma on the biofilm does not depend on the whether microorganisms are grown in a closed system (batch) or an open one (continuous culture).

**Figure 3:**
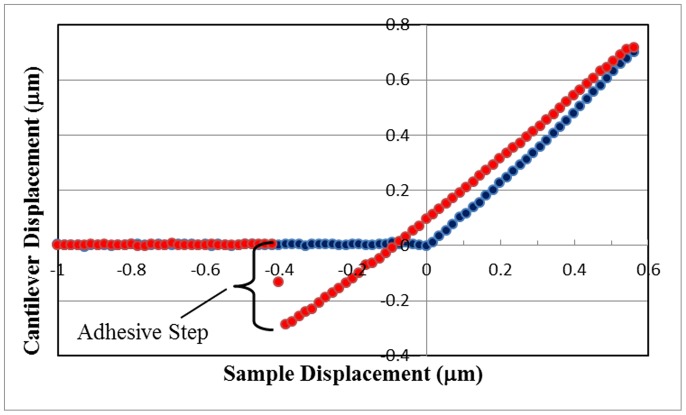
*P. aeruginosa* biofilm force-displacement curve for the control sample on a glass coupon. Data for tip-sample approach (blue circles) and tip-sample retraction (red circles) are shown. The negative displacement of the cantilever that occurred due to tip adhesion to the biofilm upon retraction is designated as the adhesive step, and is measured to be 0.288 µm for this curve.

Column (a) of Figure 2 clearly displays distinguishable bacteria together with large smooth areas of biofilm matrix presumably composed of exopolysaccharide. However, as displayed in columns (b) and (c) in the same figure, matrix areas become reduced/smaller upon exposure to plasma. For 30 minutes of plasma treatment (column (c)) no matrix areas can be seen. In fact, of the seven samples measured, matrix areas were never observed in any of the 30-minute plasma treatments. This qualitative observation suggests that plasma treatment may reduce the areas of biofilm matrix probably through oxidation/peroxidation of the exopolysaccharide by the reactive agents, mostly free radicals, present in the plasma. We hypothesized that the decrease and eventual loss of the biofilm matrix would reduce the adhesiveness of the biofilm to the surface to which it is anchored and would lead to disorganization of the tridimensional structure of the biofilm. To test this hypothesis we assessed micromechanical properties of the biofilm through force-displacement curves as described in Materials and Methods. Figure 3 shows a typical force-displacement curve for *P. aeruginosa* biofilms grown on glass coupons in continuous culture and not treated with plasma (control). The curve displays the same general features that were exhibited in all of our force-displacement curves and is similar to the ones we previously reported [10]. The tip encounters the sample surface upon approach (blue data, at the origin of the graph) and deflects upward with an increasing slope. Upon retraction (red data), the tip only roughly retraces the approaching curve with moderate hysteresis. Upon further retraction, the tip adheres to the surface until it breaks free, and the points retrace the approaching data along the negative x axis of the graph. For this study, to ascertain surface adhesion, only one section of the curve was analyzed, the height of the adhesive step upon retraction, as defined in Figure 3.

**Figure 4:**
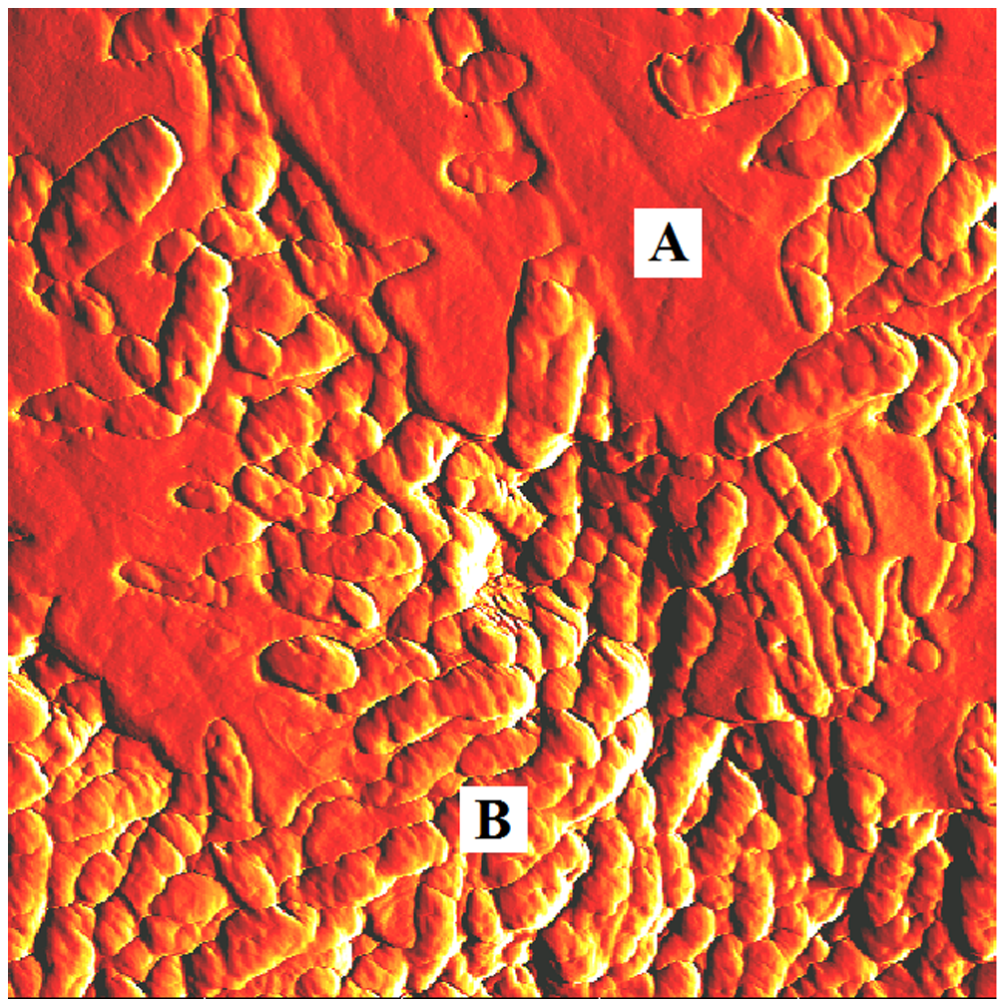
AFM images. 10×10 µm^2^ area AFM image of *P. aeruginosa* biofilm for the control sample indicating locations for obtaining force-displacement curve data. Force-displacement curves obtained at locations similar to point A were designated as areas of predominately matrix material and locations similar to point B were designated as areas of predominately bacteria.

**Figure 5:**
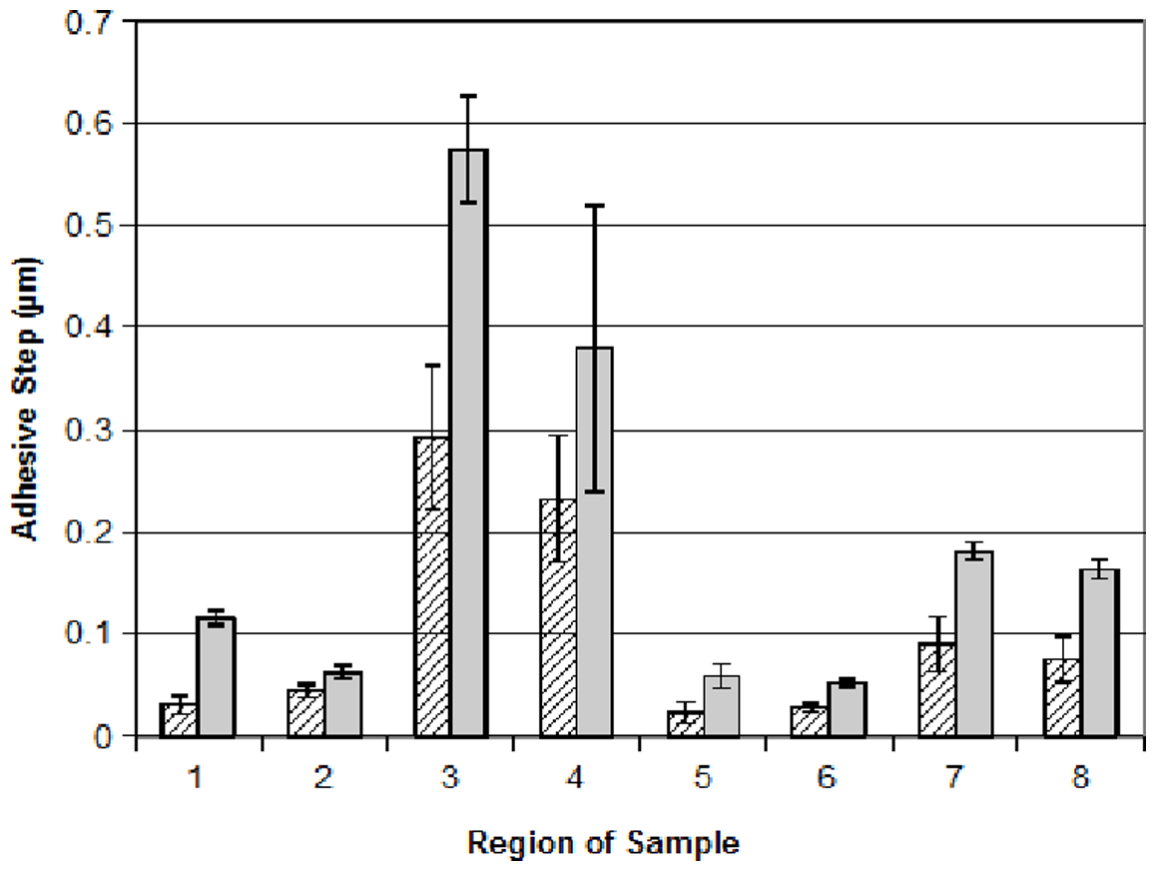
*P. aeruginosa* biofilm force-displacement curve adhesive step data for 0 minute (control) plasma-treated sample 1. The height of each bar on the graph corresponds to the mean adhesive step height of five curves obtained for each region. Error bars represent the standard error of the mean. Hatched bars and solid bars represent measurements on mostly bacteria and mostly matrix areas, respectively.

Force-displacement curves were obtained from two distinct sample locations, areas of mostly matrix material or areas of mostly bacteria, as indicated in Figure 4. Figure 5 displays adhesive step results for sample 1 obtained over a number of regions on the sample. It is apparent from this graph that there is a wide variability in adhesive step values over the various regions of the sample. Even considering this variability, for each region, there is more adhesion for the mostly matrix areas than for areas of mostly bacteria. This general result was also confirmed for the other three samples measured. For each sample, the average value of the heights of the adhesive steps was always greater when measured at mostly matrix areas than for areas of mostly bacteria.

**Figure 6:**
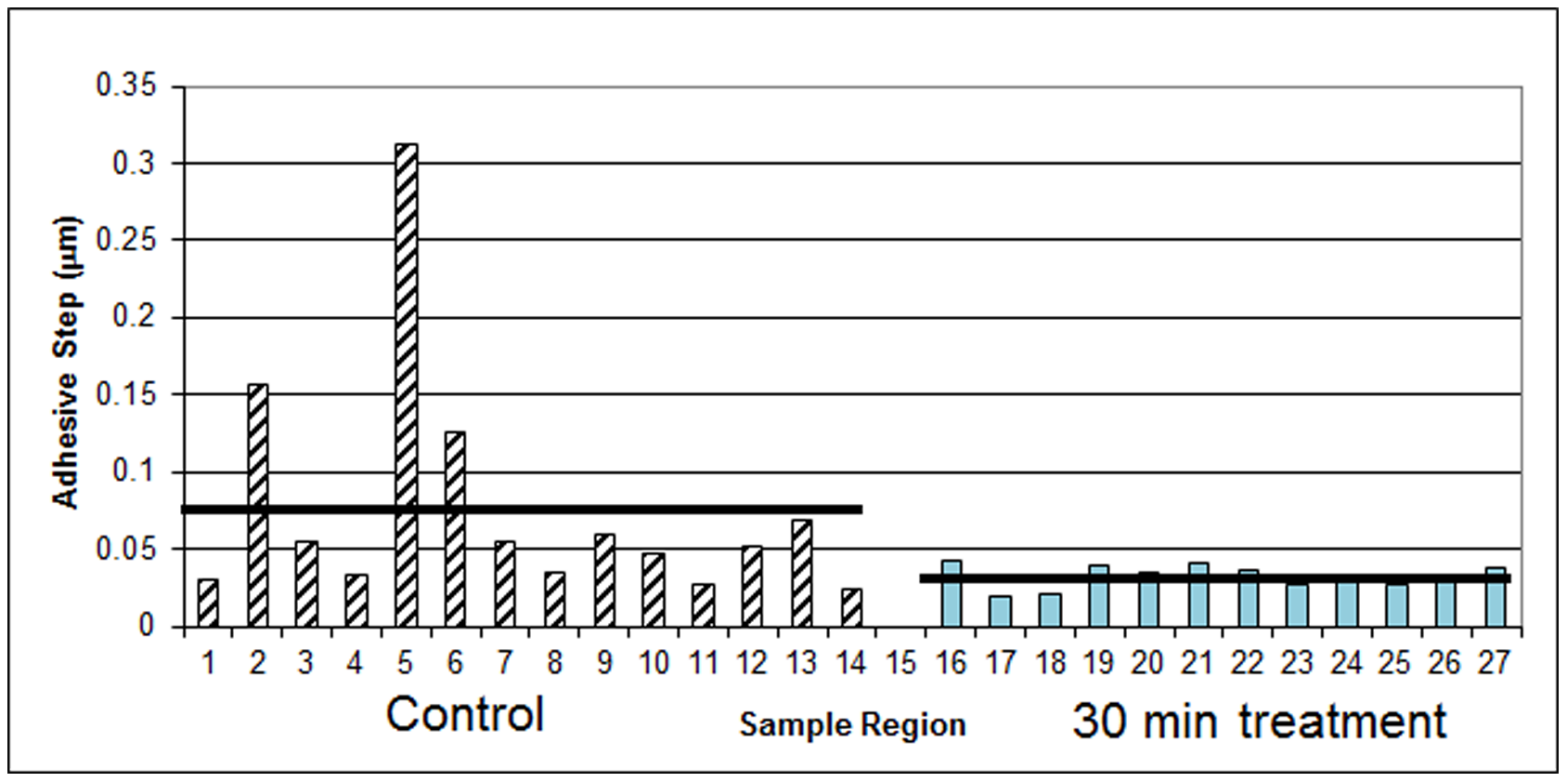
*P. aeruginosa* biofilm force-displacement curve adhesive step data (sample 3), 0 minute (control, hatched bars) and 30 minute plasma-treatment (solid bars). The height of each bar on the graph corresponds to the mean slope of five curves obtained for each region. The horizontal black bars represent the mean adhesive step values.

In addition to examining adhesion differences due to the matrix versus the bacteria, force-displacement curves were obtained on two of the samples comparing the 0 minute to the 30-minute plasma treatment. For these comparisons, curves were obtained only on areas of predominately bacteria since none of the 30-minute treatments yielded any areas of mostly matrix, as described previously. Figure 6 displays these results for one of the samples. Although there is again variability over different regions of the sample, on average, the adhesion to the plasma treated areas is significantly less than for the control. The mean adhesive step for this sample was 0.077 µm for the control and 0.032 µm for the 30-minute plasma treatment. For another sample (data not shown), the mean adhesive step was 0.030 and 0.022 µm for the control and 30 minute- plasma treatment, respectively.

Taken together with the results from about 300 images and approximately 700 force displacement curves analyzed, we can conclude that plasma treatment of *P. aeruginosa* biofilms results in little change in cell morphology for short exposure times while longer exposures results in significant loss of the biofilm structure resulting in cell death. The adhesiveness of the biofilm varies across its structure and is higher in areas with larger amounts of matrix. Plasma treatment removes or at least reduces the matrix, presumably by oxidation/peroxidation due to the presence of free radicals, and the areas of predominantly bacteria are less adhesive after the treatment.

### Virulence tests

**Figure 7:**
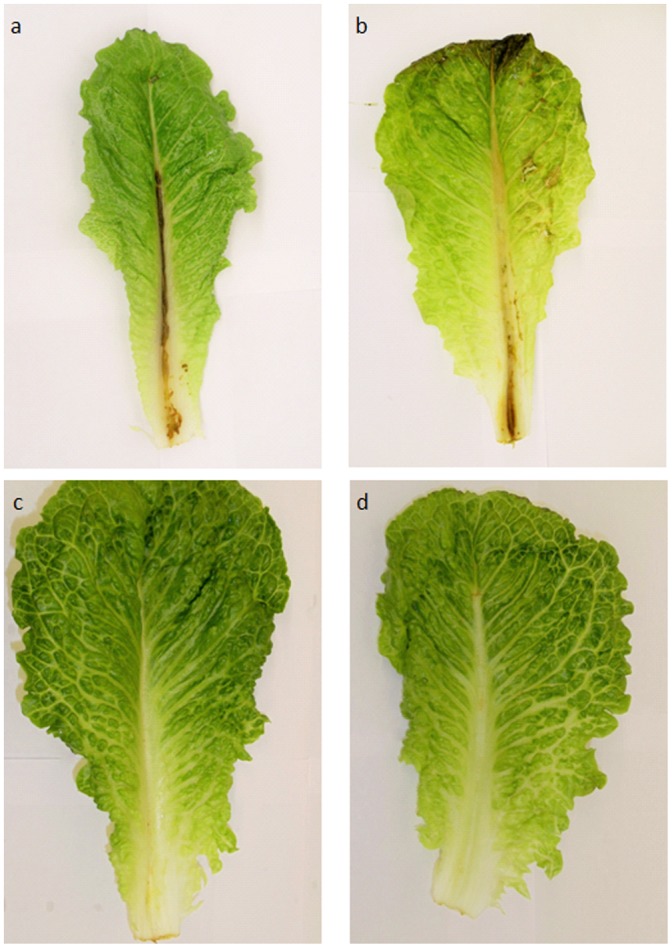
*P. aeruginosa* virulence tests on lettuce leaves. Lettuce leaves were injected with *P. aeruginosa* biofilm-forming cells treated with plasma for 0, 1 or 30 minutes (panels “a”, “b” and “c” respectively) as described in Materials and Methods. A control injected with saline (panel “d”) was included as a negative control.

*P. aeruginosa* is an opportunistic pathogen for humans but also a plant pathogen that produces tissue damage. Therefore we developed a plant assay that allowed us to test for viability. Figure 7 shows the results of the virulence tests on lettuce leaves. It is clear that the mid-vein of leaves in panels “c” and “d” show no damage whereas both the leaf treated with bacteria not subjected to plasma treatment (panel “a” positive control) or treated with plasma for one minute (panel “b”) show tissue damage. Although bacterial cells treated with plasma for one minute do not yield appreciable numbers of colonies on a petri dish, it is obvious that those cells are still not only viable but also virulent.

## Conclusions

Our results demonstrate that *P. aeruginosa* bacterial biofilms grown on glass under continuous culture can be inactivated by using gas discharge plasma. Both biofilm architecture and cell culturabilty are impacted by the plasma treatment. These results are evidence of the potential of plasma as an alternative sterilization method against biofilms.

One of the issues of more concern regarding plasma-assisted cell inactivation is that many authors still assess the lethality of plasma solely based on the number of colonies that can be counted after the treatment. However, bacterial cells can respond to one or more environmental stresses by entering a viable-but-non-culturable (VBNC) state [29] and although they are unable to produce colonies on an agarized medium they are still alive and may retain pathogenicity. The virulence assay using lettuce plants for *P. aeruginosa* biofilms indicates that cells retain viability after short exposures to plasma. Therefore, and in agreement with previously reported results from our group [10], [19], [26], viability experiments should always be carried out before drawing the conclusion that plasma is useful to kill cells based solely on measurement of culturable cells.

AFM results show that plasma treatment of *P. aeruginosa* biofilms results in little change in cell morphology for short exposure times while longer exposures results in significant loss of the biofilm structure. The adhesiveness of the biofilm varies across its structure and is higher in areas with larger amounts of matrix. Plasma treatment removes or at least reduces the matrix, presumably by oxidation/peroxidation due to the presence of free radicals, and the areas of predominantly bacteria are less adhesive after the treatment. An interesting observation from the AFM experiments is that biofilms treated with plasma for 30 minutes resulted in images that consistently had a “noisier” background than the images corresponding to the control or to 1-minute exposure to plasma. These results suggest changes in the biofilm surface chemistry that may affect the AFM tip interaction with the surface. In a previous work we studied the chemistry of the generated plasma by spectroscopy and we reported the presence of OH and NO radicals in the plasma [5]. These reactive species have direct impact on microorganisms, especially on the cell wall and cell membrane compromising their function and viability and altering the surface chemistry. It is well known that free radicals produce oxidation and peroxidation of lipids of the bacterial outer envelopes and cytoplasmic membrane. It is also possible that the exopolisaccharide that composes the biofilm matrix and anchors bacteria to the surface may become oxidized by plasma as well. Therefore, it can be speculated that the background “noise” observed in images obtained from plasma treated biofilms results from changes in the biofilm surface chemistry after plasma exposure. Further HPLC experiments will be carried out to study putative changes in the biofilm matrix exopolysaccharide.
